# Traditional Chinese Medicine for Coronary Heart Disease: Clinical Evidence and Possible Mechanisms

**DOI:** 10.3389/fphar.2019.00844

**Published:** 2019-08-02

**Authors:** Ke-Jian Zhang, Qun Zheng, Peng-Chong Zhu, Qiang Tong, Zhuang Zhuang, Jia-Zhen Zhu, Xiao-Yi Bao, Yue-Yue Huang, Guo-Qing Zheng, Yan Wang

**Affiliations:** ^1^Department of Cardiology, the Second Affiliated Hospital and Yuying Children’s Hospital of Wenzhou Medical University, Wenzhou, China; ^2^Department of Neurology, the Second Affiliated Hospital and Yuying Children’s Hospital of Wenzhou Medical University, Wenzhou, China

**Keywords:** Traditional Chinese medicine, coronary heart disease, high-quality randomized controlled trials, clinical evidence, possible mechanisms, systematic review

## Abstract

Coronary heart disease (CHD) remains a major cause of mortality with a huge economic burden on healthcare worldwide. Here, we conducted a systematic review to investigate the efficacy and safety of Chinese herbal medicine (CHM) for CHD based on high-quality randomized controlled trials (RCTs) and summarized its possible mechanisms according to animal-based researches. 27 eligible studies were identified in eight database searches from inception to June 2018. The methodological quality was assessed using seven-item checklist recommended by Cochrane Collaboration. All the data were analyzed using Rev-Man 5.3 software. As a result, the score of study quality ranged from 4 to 7 points. Meta-analyses showed CHM can significantly reduce the incidence of myocardial infarction and percutaneous coronary intervention, and cardiovascular mortality (P < 0.05), and increase systolic function of heart, the ST-segment depression, and clinical efficacy (P < 0.05). Adverse events were reported in 11 studies, and CHMs were well tolerated in patients with CHD. In addition, CHM exerted cardioprotection for CHD, possibly altering multiple signal pathways through anti-inflammatory, anti-oxidation, anti-apoptosis, improving the circulation, and regulating energy metabolism. In conclusion, the evidence available from present study revealed that CHMs are beneficial for CHD and are generally safe.

## Introduction

Coronary heart disease (CHD) incurs a huge economic burden on healthcare and society (Dunbar et al., 2018). According to the epidemiological data from 1990 to 2013, 92.94 million people were suffering from this disease, which eventually led to 8.1 million deaths (Murray et al., 2015; Roth et al., 2015). Current treatments for CHD include coronary revascularization, drug intervention, risk factor control, cardiac rehabilitation, and lifestyle improvement (Arslan et al., 2018). Among them, percutaneous coronary intervention (PCI) and coronary artery bypass grafting are the most effective (Roffi et al., 2016). However, PCI is mainly for the treatment of locally severe stenotic vessels and has limited therapeutic effect on extensive coronary stenosis and microcirculation lesions (Heusch and Gersh, 2017). Meanwhile, the prognosis of patients treated with PCI is sometimes not ideal because myocardial ischemia/reperfusion injury, no reflow, coronary dissection, stent thrombosis, and acute coronary occlusion still exist (Hausenloy and Yellon, 2013; Arslan et al., 2018). Although the technology of coronary intervention is still improving and conventional medicine is constantly updating, novel treatments that can stabilize arterial plaque, improve microcirculation, and angina symptoms; prevent acute myocardial infarction; delay the development of ischemic cardiomyopathy; ultimately reduce PCI; and improve prognosis are urgently needed.

Traditional Chinese medicine (TCM) includes herbal medicine (CHM), acupuncture, and other non-pharmacological therapies, which is a holistic approach to health and healing (Xu et al., 2013). CHM has been used to treat CHD for thousands of years, and in modern time, many claimed randomized controlled trials (RCT) have reported some TCM Fufang exerted the cardioprotective function (Han et al., 2008; Gao et al., 2010; Chung et al., 2013; Liu et al., 2013). However, most of these studies are poor methodological quality, leading that there is still insufficient evidence to support routine use of CHMs for CHD. Thus, the Cochrane group guidelines for clinical reviews may exclude the ‘‘not-so-good’’ studies (Chan et al., 2012). In addition, in a TCM reviewing process, researchers may need to include such high-quality studies about a medical certain issue to identify current problems and areas worthy of improvement for its future development (Chan et al., 2012). Thus, we performed a systematic review to assess the efficacy and safety of CHM for CHD according to high-quality studies with at least four domains of “yes” in Cochrane risk of bias (RoB) tool (Li et al., 2015).

## Methods

### Search Strategy and Study Selection

Studies estimating the efficacy of CHMs in patients with CHD were systematically searched from EMBASE, PubMed, Cochrane Library, Wangfang database, China National Knowledge Infrastructure (CNKI), VIP database (VIP), and China Biology Medicine disc (CBM) from inception to the end of June 2018. The key words were used as follows: “coronary disease OR acute coronary syndrome OR myocardial infarction OR myocardial ischemia” AND “herb OR traditional Chinese medicine OR Chinese Materia Medica.” Moreover, reference lists of potential articles were searched for relevant studies.

### Inclusion and Exclusion Criteria

The inclusion criteria were prespecified as follows: (1) RCTs that investigated the efficacy and safety of CHM for CHD were included. Quasi-randomized trials, such as those in which patients were allocated according to date of birth and order of admission number, were excluded. If a three-arm design was used in a study, we extracted data only for the group(s) involving CHM and the control group(s). (2) All participants were patients with a diagnosis of CHD based on one of the following criteria: (1) The guideline of unstable or stable angina from Chinese cardiovascular association in different years, (2) the guideline of unstable or stable angina from American College of Cardiology (ACC) or American Heart Association (AHA) or European Society of Cardiology (ESC) or World Health Organization (WHO) in different years, (3) be diagnosed by coronary angiography, (4) patients after PCI, and (5) diagnostic criteria made by other authors with comparable definitions were also used. (3) The treatment interventions included CHMs used as monotherapies or adjunct with conventional medicine (i.e., antiplatelet, stable plaque, control ventricular rate) or supportive treatment (i.e., nutrition support, exercise therapy, psychotherapy). Interventions for control group were restricted to no intervention, placebo, conventional medicine, and supportive treatment. Studies comparing a CHM agent with another CHM agent were excluded. (4) The primary outcome measures were the incidence of myocardial infarction and/or the incidence of PCI and/or cardiovascular mortality and/or the level of ST-segment depression and/or indicators which represent systolic and diastolic function of the heart in cardiac ultrasound. The secondary outcome measures were clinical effective rating, and the safety of co-administration of CHM. The exclusion criteria were prespecified as follows: (1) no predetermined outcome index; (2) compared or combined with other Chinese herb medicine; (3) not randomized, double-blind, placebo-controlled designed; (4) no control group; and (5) double publication.

### Data Extraction

Two authors independently reviewed each included study and extracted following aspects of details: (1) name of first author, year of publication; (2) diagnostic criteria; (3) detail information of participants for each study, including sample size, gender composition, and mean age; (4) detail information of treatment and control group, including therapeutic drug dosage, method of administration, and duration of treatment; and (5) outcome measures and intergroup differences. The data of predetermined primary and secondary outcomes were extracted for further qualitative and quantitative syntheses. We made efforts to contact authors for further information when some records’ published data were only in graphical format or not in the publication. And the numerical values were measured from the graphs by digital ruler software when response was not received from authors.

### Risk of Bias in Individual Studies

The methodological quality of each included study was evaluated by two authors with the seven-item checklist recommended by Cochrane Collaboration (Higgins and Green, 2012). Only RCTs with a cumulative score of at least four points were included in our systematic review. Any disagreements from two authors were dealt with through discussion with the corresponding author (GQZ).

### Statistical Analysis

The statistical analysis was conducted *via* RevMan version 5.3. A fixed-effects model (FEM) or random-effects model (REM) was conducted to analyze pooled effects. When the outcome measurements in all included studies in meta-analysis were based on the same scale, weighted mean difference (WMD) with 95% confidence intervals was calculated as a summary statistic, otherwise standard mean difference (SMD) was calculated. Heterogeneity between study results was investigated based on a standard chi-square test and I^2^ statistic. A fixed-effects model (I^2^ < 50%) or a random-effects model (I^2^ > 50%) was used depending on the value of I^2^. Funnel plots were used to visually estimate publication bias. A probability value 0.05 was considered statistically significant.

### CHM Composition and Possible Mechanisms of Active Ingredients

Specific herbs in the CHM formulae were recorded. The frequency of use for particular herb was calculated and those used at a high frequency that are described in detail. Animal-based mechanism studies of active ingredients from frequently used herbs were searched. The following information was recorded for such studies: identity of active ingredients and their herbal sources, suggested mechanisms and implicated signaling pathways, first author’s name and publication year of the citation, and structure of active ingredients.

## Results

### Study Selection

**Figure 1:**
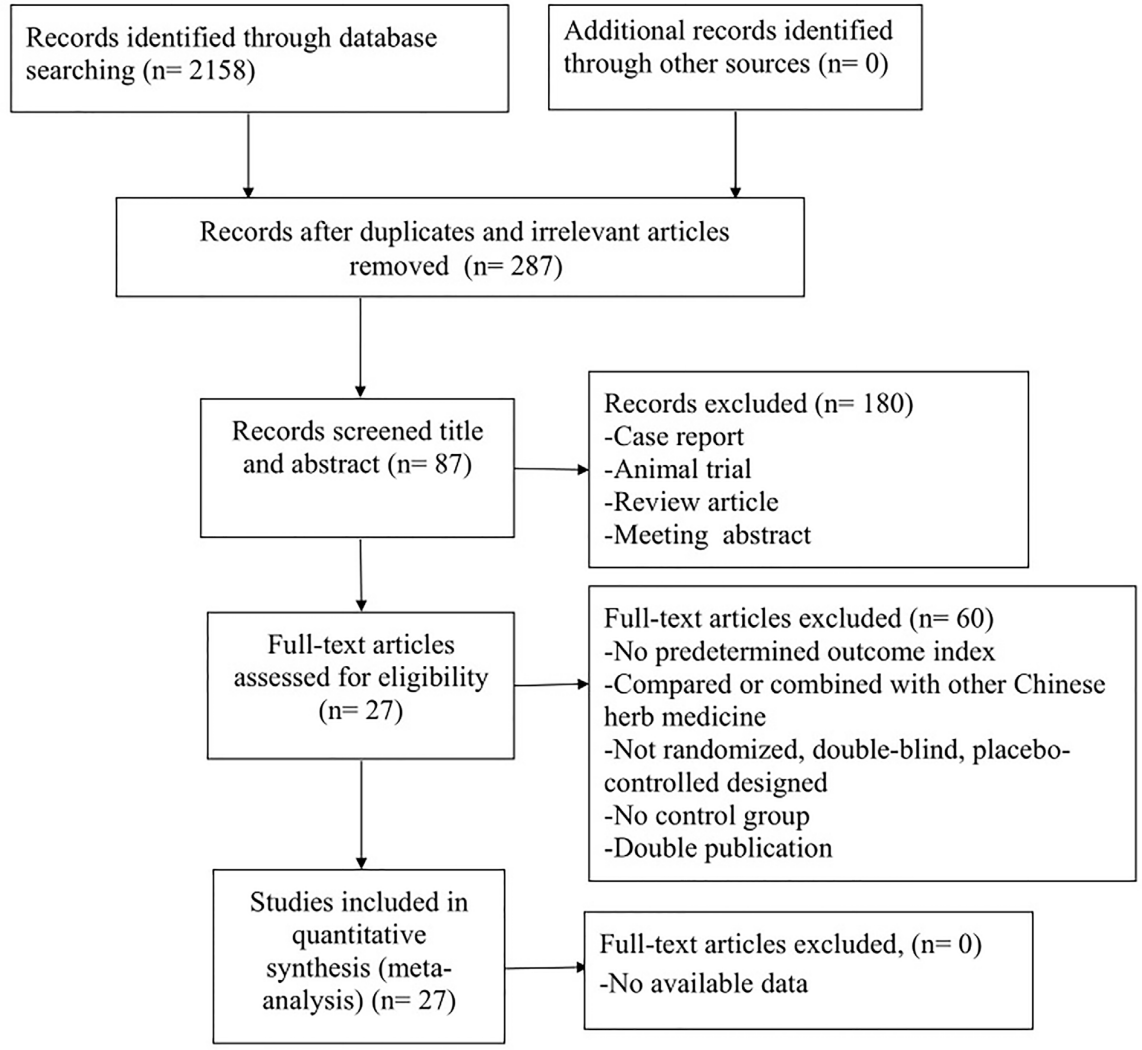
Summary of the process for identifying candidate studies.

A total of 2,158 studies were retrieved after systematical searches from the database, of which 287 were reduplicated and irrelevant studies. After screening title and abstract, 180 were excluded because they were: (1) animal trial, (2) case report, (3) review article, and (4) meeting abstract. After reviewing the full text of the remaining 87 articles, 60 studies were excluded if: (1) no predetermined outcome index; (2) compared or combined with other CHM; (3) not randomized, double-blind, and placebo-controlled designed; (4) no control group; (5) double publication; and (6) data of result was not available. Ultimately, 27 studies with Cochrane RoB score ≧4 (Lu et al., 2006; Qiao et al., 2006; Lu et al., 2008; Cheng et al., 2009; Chu et al., 2010; Qiu et al., 2009; Wang et al., 2009; Zhang et al., 2010; Shang et al., 2011; Mo et al., 2012; Wang S. H. et al., 2012; Wang Y. G. et al., 2012; Chen et al., 2013; Shang et al., 2013; Hu et al., 2014; Liu et al., 2014; Lu et al., 2014; Sun, 2014; Xu et al., 2014; Xu et al., 2015; Zhang et al., 2015; Duan et al., 2016; Mao et al., 2016; Wang et al., 2016; Zhu et al., 2016; Wang et al., 2017; Yang et al., 2017) were selected (**Figure 1**).

### Characteristics of Included Studies

17 studies (Lu et al., 2006; Qiao et al., 2006; Cheng et al., 2009; Chu et al., 2010; Qiu et al., 2009; Mo et al., 2012; Wang S. H. et al., 2012; Wang Y. G. et al., 2012; Chen et al., 2013; Hu et al., 2014; Liu et al., 2014; Lu et al., 2014; Sun, 2014; Xu et al., 2014; Xu et al., 2015; Duan et al., 2016; Zhu et al., 2016; Wang et al., 2017; Yang et al., 2017) were published in Chinese and 10 studies (Lu et al., 2008; Wang et al., 2009; Zhang et al., 2010; Chu et al., 2010; Shang et al., 2011; Shang et al., 2013; Xu et al., 2015; Zhang et al., 2015; Mao et al., 2016; Wang et al., 2016) in English between 2006 and 2017. All studies were conducted in China. The sample size of the included studies ranged from 57 to 4,870 with a total of 11,732 participants, including 5,916 patients in treatment groups and 5,816 patients serving as controls. Of 27 included studies, 18 studies (Cheng et al., 2009; Qiu et al., 2009; Wang et al., 2009; Shang et al., 2011; Mo et al., 2012; Wang S. H. et al., 2012; Shang et al., 2013; Hu et al., 2014; Liu et al., 2014; Sun, 2014; Xu et al., 2014; Zhang et al., 2015; Duan et al., 2016; Wang et al., 2016; Zhu et al., 2016; Wang et al., 2017; Yang et al., 2017) were based on patients with angina pectoris of CHD and nine studies (Lu et al., 2006; Qiao et al., 2006; Lu et al., 2008; Chu et al., 2010; Zhang et al., 2010; Chen et al., 2013; Lu et al., 2014; Xu et al., 2015; Mao et al., 2016) were based on patients with acute coronary syndrome. Comparisons of CHM plus a conventional treatment (i.e., antiplatelet, stable plaque, control ventricular rate) *versus* a conventional treatment were conducted in 26 trials, and comparisons of CHM versus a placebo were performed in one trial (Hu et al., 2014). The CHMs were administered orally (i.e., tablets, capsules, granules, or decoction). The duration of follow-up was varied from 4 weeks to 4.5 years. All studies accounted for baseline comparability. The incidence of myocardial infarction (MI) was utilized as outcome measure in 10 studies (Lu et al., 2006; Lu et al., 2008; Wang et al., 2009; Shang et al., 2011; Shang et al., 2013; Lu et al., 2014; Sun, 2014; Xu et al., 2015; Mao et al., 2016; Wang et al., 2016), the incidence of PCI in five studies (Lu et al., 2006; Lu et al., 2008; Wang et al., 2009; Shang et al., 2013; Wang et al., 2016), cardiovascular mortality in seven studies (Lu et al., 2008; Wang et al., 2009; Shang et al., 2013; Sun, 2014; Xu et al., 2015; Mao et al., 2016; Wang et al., 2016), left ventricular ejection fraction (LVEF) in five studies (Qiao et al., 2006; Qiu et al., 2009; Chen et al., 2013; Sun, 2014; Mao et al., 2016), the ventricular wall motion score in two studies (Qiao et al., 2006; Chen et al., 2013), and the level of ST-segment elevation in three studies (Chu et al., 2010; Hu et al., 2014; Sun, 2014). The efficiency of angina improved was reported in 12 studies (Lu et al., 2006; Chu et al., 2010; Shang et al., 2011; Mo et al., 2012; Wang S. H. et al., 2012; Hu et al., 2014; Lu et al., 2014; Sun, 2014; Zhang et al., 2015; Duan et al., 2016; Zhu et al., 2016; Yang et al., 2017), the usage of nitroglycerin in two studies (Sun, 2014; Xu et al., 2014), low-density lipoprotein (LDL) in four studies (Lu et al., 2008; Wang Y. G. et al., 2012; Zhu et al., 2016; Wang et al., 2017), hypersensitive C-reactive protein (hsCRP) in two studies (Mo et al., 2012; Wang S. H. et al., 2012), the degree of coronary artery stenosis in two studies (Lu et al., 2006; Yang et al., 2017), and the rate of coronary restenosis in two studies (Shang et al., 2011; Lu et al., 2014). The overall characteristics of included studies are shown in **Table 1**.

**Table 1:** Characteristics of the 27 included studies.

Study (years)	Diagnostic criteria	Number of participants (male/female), mean age (years)		Interventions		Conventional medicine or basic treatment	Duration of treatment	Outcome index	Intergroup differences
		Trial	Control	Trial	Control				
Lu et al., 2006	After PCI	60 58.94 ± 10.79	58 57.1 ± 9.81	Xiongshao capsule (0.5g, tid, p.o).	Placebo	Clopidogrel, aspirin; atorvastatin, low molecular weight heparin	6 months	1. Cardiovascular mortality 2. The rate of coronary restenosis 3. The degree of coronary artery stenosis 4. The efficiency of angina pectoris 5. Myocardial infarction rate 6. The incidence of PCI	1. P < 0.05 2. P < 0.05 3. P < 0.05 4. P < 0.05 5. P < 0.05 6. P < 0.05
Qiao et al., 2006	After PCI	30 (17/13) 64.0 ± 11.2	29 (18/11) 65.7 ± 12.2	Tongguan capsule (three doses, tid, p.o).	Placebo	Anticoagulant, antiplatelet, anti-infection	1 month	1. LVEF 2. The ventricular wall motion score 3. Survey of angina pectoris in Seattle	1. P < 0.05 2. P < 0.05 3. P < 0.05
Lu et al., 2008	Documented previous myocardial infarction	2,429 58.35 ± 9.02	2,441 58.35 ± 9.02	Xuezhikang capsule (0.6g, bid, p.o).	Placebo	Other drugs that do not affect blood lipids	4.5 years	1. Myocardial infarction rate 2. Cardiovascular mortality 3. The incidence of PCI 4. TC 5. TG 6. HDL-C 7. LDL-C	1. P < 0.05 2. P < 0.05 3. P < 0.05 4. P < 0.01 5. P < 0.01 6. P < 0.01 7. P < 0.01
Cheng et al., 2009	The guideline of chronic stable angina from China, 2007	41 (37/4) 49.97 ± 6.19	41 (39/2) 51.12 ± 7.33	Qingre Quyu granule (6g, bid, p.o).	Placebo	Aspirin, bisoprolol fumarate, isosorbide dinitrate sustained release tablets	25 weeks	1. Number of atherosclerotic plaques 2. Arterial plaque score 3. Intima thickness of carotid artery 4. hsCRP	1. P < 0.05 2. P < 0.05 3. P < 0.05 4. P < 0.05
Qiu et al., 2009	The guideline of acute myocardial infarction from China, 2001	51 (45/6) 57.82 ± 10.23	52 (44/8) 55.79 ± 11.06	Compound *Salvia* tablet and Xinyue capsule	Placebo	Antiplatelet agents, anticoagulant, β blocker, angiotensin converting enzyme inhibitor, nitrates, and lipid-regulating drugs	3 months	1. LVEF	1. P < 0.05
Wang et al., 2009	The guideline of unstable angina pectoris from ACC/AHA USA, 2002	32 (12/20) 61.65 ± 8.15	31 (13/18) 64.47 ± 9.21	Shenshao tablet (0.3g, qd, p.o).	Placebo	Aspirin, isosorbide, mononitrate, simvastatin, Benner Pury, amlodipine, metoprolol	4 weeks	1. Frequency of angina pectoris 2. Seattle score 3. The incidence of PCI 4. Acute myocardial infarction rate	1. P < 0.05 2. P < 0.05 3. P > 0.05 4. P > 0.05
Chu et al., 2010	After PCI	28 (18/10); 61.7 ± 9.6	29 (20/9); 58.8 ± 8.9	Xuefu Zhuyu capsule	Placebo	Clopidogrel, aspirin, low molecular weight heparin, metoprolol tartrate, atorvastatin	4 weeks	1. The efficiency of angina pectoris 2. Electrocardiogram curative effect 3. Survey of angina pectoris in Seattle	1. P < 0.05 2. P < 0.05 3. P < 0.05
Zhang et al., 2010	The guideline of segment elevation myocardial infarction from WHO	108 (92/16) 58.5 ± 10.6	111 (96/15) 57.6 ± 11.2	Tongxinluo (2.08g, qd, p.o).	Placebo	Aspirin, clopidogrel	180 days	1. The incidence of no reflow of myocardium	1. P = 0.0031
Shang et al., 2011	The guideline of stable angina pectoris from WHO	73 (50/23) 67.79 ± 4.77	79 (52/27) 66.7 ± 4.16	Chuangxiongol (250mg, tid, p.o).	Placebo	Aspirin, tilopidine, diltiazem, nitroglycerin, heparin	6 months	1. Restenosis rate 2. The efficiency of angina pectoris 3. Cardiovascular mortality 4. Acute myocardial infarction rate 5. The incidence of PCI	1. P > 0.05 2. P < 0.01 3. P > 0.05 4. P > 0.05 5. P > 0.05
Mo et al., 2012	The guideline of criteria for the naming and diagnosis of ischemic heart disease	60 (43/17) 67.15 ± 4.87	60 (42/18) 66.22 ± 5.12	Yixin Mai granule (one dose, tid, p.o).	Placebo	Isosorbide, metoprolol, fosinopril, aspirin	4 weeks	1. hsCRP 2. IL-6 3. IL-18 4. The efficiency of angina pectoris	1. P < 0.01 2. P < 0.01 3. P < 0.01 4. P = 0.037
Wang S. H. et al., 2012	The guideline of unstable angina pectoris from ACC/AHA USA, 2002	33 (26/7) 60.2 ± 9	33 (25/8) 62.7 ± 7.1	Tablets of betel (1.5g, bid, p.o).	Placebo	Aspirin, simvastatin, isosorbide, dinitrate	28 days	1. The efficiency of angina pectoris 2. Electrocardiogram efficiency 3. Nitroglycerin consumption 4. hsCRP 5. sCD40L	1. P < 0.05 2. P > 0.05 3. P < 0.05 4. P < 0.05 5. P < 0.05
Wang Y. G. et al., 2012	The guideline of chronic stable angina from China, 2007	76	72	Double ginseng capsule and Tongguan capsule (four doses, tid, p.o).	Placebo	Original treatment	6 months	1. TG 2. TC 3. HDL-C 4. LDL-C	1. P < 0.05 2. P > 0.05 3. P < 0.05 4. P < 0.05
Chen et al., 2013	After PCI	30 (17/13) 65.5 ± 7.5	30 (16/14) 63.8 ± 6.3	Tongguan capsule (three doses, tid, p.o).	Placebo	Clopidogrel, aspirin, low molecular weight heparin	3 months	1. LVEF 2. The ventricular wall motion score 3. The number of endothelial progenitor cell in peripheral blood	1. P < 0.05 2. P < 0.05 3. P < 0.05
Shang et al., 2013	The guideline of chronic stable angina from China, 2004	1,746 (1,191/555) 58.35 ± 9.02	1,759 (1,260/499) 58.28 ± 8.99	QSYQ (0.5g, tid, p.o).	Placebo	Antihypertensive drugs, hypoglycemic agent, lipid-lowering medicine	12 months	1. Cardiovascular mortality 2. Myocardial infarction rate 3. The incidence of PCI	1. P > 0.05 2. P > 0.05 3. P > 0.05
Hu et al., 2014	The guideline of chronic stable angina from China, 2007	192 57.82 ± 10.23	99 57.82 ± 10.23	Reachable film (three doses, tid, p.o).	Placebo	NM	4 weeks	1. The efficiency of angina pectoris 2. The total curative effect of TCM Syndrome 3. Electrocardiogram efficiency	1. P < 0.05 2. P < 0.05 3. P < 0.05
Liu et al., 2014	The guideline of chronic stable angina from China, 2007	120 59.21 ± 7.92	120 60.64 ± 7. 69	Chek Shincen Tongxin granule	Placebo	Aspirin, atorvastatin	4 weeks	1. Body limitation 2. Stable state of angina pectoris 3. Episodes of angina pectoris 4. Satisfaction with treatment 5. The degree of understanding of disease 6. Electrocardiogram efficiency	1. P < 0.05 2. P < 0.05 3. P < 0.05 4. P < 0.05 5. P < 0.05 6. P < 0.05
Lu et al., 2014	After PCI	90 (48/42) 60.2 ± 6.9	90 (46/44) 61.8 ± 7.2	Tongxinluo capsule (three doses, tid, p.o).	Placebo	Original treatment	12 months	1. The incidence of coronary restenosis 2. The degree of coronary restenosis 3. The efficiency of angina pectoris 4. Myocardial infarction rate 5. Cardiovascular mortality	1. P < 0.05 2. P < 0.01 3. P = 0.04 4. P = 0.19 5. P > 0.05
Sun, 2014	The guideline of chronic unstable angina from China, 2007	64 (42/22) 70.81 ± 10.76	64 (44/20) 69.8 ± 10.98	Musk Baoxin pill (two doses, tid, p.o).	Placebo	Isosorbide dinitrate tablets, atorvastatin, thiazepine, enteric aspirin	6 months	1. The efficiency of angina pectoris 2. Myocardial infarction rate 3. Cardiovascular mortality 4. Nitroglycerin consumption 5. Electrocardiogram efficiency 6. LVEF	1. P < 0.05 2. P < 0.05 3. P > 0.05 4. P < 0.01 5. P < 0.05 6. P < 0.05
Xu et al., 2014	The guideline of unstable angina pectoris from ACC/AHA USA, 2002	55 (29/26) 69.47 ± 8	59 (33/26) 70.41 ± 8.6	Shenzhu Guanxin recipe (12g, qd, p.o).	Placebo	Conventional western medicine (unspecified)	12 weeks	1. The efficiency of angina pectoris 2. The duration of angina pectoris 3. Total use of nitroglycerin 4. The degree of physical activity induced by angina pectoris 5. The degree of angina pectoris	1. P < 0.05 2. P < 0.05 3. P < 0.05 4. P < 0.05 5. P < 0.01
Xu et al., 2015	After PCI	113 (86/27) 70.35 ± 9.61	74 (51/23) 68.08 ± 10.38	Shenzhu Guanxin recipe	Placebo	Aspirin, ticplopidine, diltiazem, glyceryl, trinitrate, heparin	3 months	1. Angina pectoris score 2. Cardiovascular mortality 3. Myocardial ischemia rate	1. P = 0.66 2. P = 0.33 3. P = 0.63
Zhang et al., 2015	The guideline of unstable angina pectoris from ACC/AHA USA, 2002	119 (56/63) 59.46 ± 6.524	120 (52/68) 58.82 ± 7.061	Wufuxinnaoqing capsules	Placebo	Antiplatelet, aggregation, ACEI or ARB, statin two hydrogen arsenide	12 weeks	1. The efficiency of angina pectoris 2. Nitroglycerin consumption	1. P < 0.01 2. P < 0.01
Duan et al., 2016	The guideline of chronic stable angina from China, 2007	64 (38/26) 59.7 ± 6.34	67 (47/20) 60.7 ± 6.44	Live heart pill (two doses, tid, p.o).	Placebo	Conventional western medicine (unspecified)	8 weeks	1. Symptom score of angina pectoris 2. Nitroglycerin consumption 3. Electrocardiogram plate movement 4. Seattle scale 5. Syndromes of traditional Chinese Medicine 6. hsCRP 7. Blood lipid	1. P < 0.01 2. P < 0.01 3. P < 0.01 4. P < 0.01 5. P < 0.01 6. P > 0.05 7. P > 0.05
Mao et al., 2016	After PCI	42 67.54 ± 8.39	41 68.38 ± 10.41	Danlou tablet	Placebo	Conventional western medicine (unspecified)	90 days	1. Left ventricular end diastolic volume index 2. End systolic volume index of left ventricle 3. LVEF 4. Cardiovascular mortality 5. Myocardial infarction rate	1. P < 0.001 2. P < 0.001 3. P < 0.001 4. P < 0.05 5. P < 0.05
Wang et al., 2016	The guideline of unstable angina pectoris from ACCF/AHA USA, 2007	109 (72/37) 62.89 ± 9.23	110 (74/36) 63.89 ± 10.03	Danlou tablet (4.5g, qd, p.o).	Placebo	Antiplatelet, aggregation, anticoagulant, lipid-lowering, improvement of myocardial, remodeling, step-down	90 days	1. Cardiovascular mortality 2. Myocardial infarction rate 3. Reconstructive rate of blood vessels 4. Troponin 5. hsCRP	1. P > 0.05 2. P = 0.04 3. P > 0.05 4. P > 0.05 5. P > 0.05
Zhu et al., 2016	The guideline of chronic stable angina from China, 2007	76 (48/28) 51.8 ± 1.6	74 (46/28) 51. 5 ± 1. 4	Traditional Chinese medicine prescription (10 mg, tid, p.o).	Placebo	Isosorbide, aspirin, atorvastatin	4 weeks	1. The efficiency of angina pectoris 2. Electrocardiogram efficiency 3. TG 4. TC 5. HDL-C 6. LDL-C 7. TCM syndrome score	1. P < 0.05 2. P < 0.05 3. P < 0.05 4. P < 0.05 5. P < 0.05 6. P < 0.05 7. P < 0.05
Wang et al., 2017	The guideline of unstable angina pectoris from ACC/AHA USA, 2011	40 (17/23) 70.68 ± 6.87	40 (21/19) 71.65 ± 4.32	Xuesaitong soft capsule (0.66g, bid, p.o).	Placebo	Conventional western medicine (unspecified)	4 weeks	1. TC 2. TG 3. HDL 4. LDL 5. Survey of angina pectoris in Seattle	1. P < 0.05 2. P < 0.05 3. P < 0.05 4. P < 0.05 5. P < 0.05
Yang et al., 2017	The guideline of chronic stable angina from China, 2014	33 (20/13) 61.18 ± 6.61	33 (21/12) 61.03 ± 7.51	Coronary Ningtong prescription	Placebo	Aspirin enteric-coated tablets, simvastatin tablets, isosorbide mononitrate, metoprolol	24 weeks	1. Coronary stenosis 2. The efficiency of angina pectoris	1. P < 0.05 2. P < 0.05

PCI, percutaneous coronary intervention; LVEF, left ventricular ejection fraction; hsCRP, high sensitive C reactive protein; TC, total cholesterol; TG, total glycerol three fat; HDL, high density lipoprotein; LDL, low density lipoprotein; STEMI, segment elevation myocardial infarction; WHO, world health organization; NM, not mention; QSYQ, Qi-Shen-Yi-Qi dripping pills.

### Study Quality

The quality score of study ranged from 4 to 7 in a total of 7 points. Of which, six studies (Lu et al., 2006; Chu et al., 2010; Wang et al., 2009; Liu et al., 2014; Xu et al., 2015; Wang et al., 2016) got 7 points, three studies (Zhang et al., 2015; Duan et al., 2016; Mao et al., 2016) got 6 points, 15 studies (Qiao et al., 2006; Lu et al., 2008; Cheng et al., 2009; Qiu et al., 2009; Zhang et al., 2010; Shang et al., 2011; Mo et al., 2012; Wang S. H. et al., 2012; Chen et al., 2013; Shang et al., 2013; Hu et al., 2014; Xu et al., 2014; Zhu et al., 2016; Wang et al., 2017) got 5 points, and three studies (Lu et al., 2014; Sun, 2014; Yang et al., 2017) got 4 points. All 27 included studies had random allocation, including 10 (Qiao et al., 2006; Cheng et al., 2009; Qiu et al., 2009; Mo et al., 2012; Wang S. H. et al., 2012; Chen et al., 2013; Lu et al., 2014; Sun, 2014; Zhu et al., 2016) in which a random number table was used, eight (Lu et al., 2006; Chu et al., 2010; Wang et al., 2009; Shang et al., 2011; Wang Y. G. et al., 2012; Liu et al., 2014; Duan et al., 2016; Wang et al., 2017) that employed a computer generated random sample set, three (Xu et al., 2015; Zhang et al., 2015; Wang et al., 2016) that applied block randomization, and six (Lu et al., 2008; Zhang et al., 2010; Shang et al., 2013; Hu et al., 2014; Xu et al., 2014; Mao et al., 2016) that stated that randomization was used without providing methodological details. Of the 27 included studies, all studies reported blinding of participants and personnel and withdraw bias. Additionally, nine studies (Lu et al., 2006; Chu et al., 2010; Wang et al., 2009; Liu et al., 2014; Xu et al., 2015; Zhang et al., 2015; Duan et al., 2016; Mao et al., 2016; Wang et al., 2016) reported using allocation concealment; eight studies (Lu et al., 2006; Chu et al., 2010; Wang et al., 2009; Liu et al., 2014; Xu et al., 2015; Zhang et al., 2015; Duan et al., 2016; Wang et al., 2016) applied blinding specifically during outcome measure assessment, and 22 studies (Lu et al., 2006; Qiao et al., 2006; Lu et al., 2008; Cheng et al., 2009; Chu et al., 2010; Qiu et al., 2009; Wang et al., 2009; Zhang et al., 2010; Shang et al., 2011; Mo et al., 2012; Wang S. H. et al., 2012; Chen et al., 2013; Shang et al., 2013; Hu et al., 2014; Liu et al., 2014; Xu et al., 2014; Xu et al., 2015; Mao et al., 2016; Wang et al., 2016; Zhu et al., 2016; Wang et al., 2017) reported selective reporting. No study provided sample size estimation information. The methodological quality is concluded in **Table 2**.

**Table 2:** Quality assessment of included studies.

Study	A	B	C	D	E	F	G	Total
Lu et al., 2006	1	1	1	1	1	1	1	7
Qiao et al., 2006	1	0	1	0	1	1	1	5
Lu et al., 2008	1	0	1	0	1	1	1	5
Chu et al., 2010	1	1	1	1	1	1	1	7
Cheng et al., 2009	1	0	1	0	1	1	1	5
Qiu et al., 2009	1	0	1	0	1	1	1	5
Wang et al., 2009	1	1	1	1	1	1	1	7
Zhang et al., 2010	1	0	1	0	1	1	1	5
Shang et al., 2011	1	0	1	0	1	1	1	5
Mo et al., 2012	1	0	1	0	1	1	1	5
Wang S. H. et al., 2012	1	0	1	0	1	1	1	5
Wang Y. G. et al., 2012	1	0	1	0	1	1	1	5
Chen et al., 2013	1	0	1	0	1	1	1	5
Shang et al., 2013	1	0	1	0	1	1	1	5
Lu et al., 2014	1	0	1	0	1	0	1	4
Hu et al., 2014	1	0	1	0	1	1	1	5
Liu et al., 2014	1	1	1	1	1	1	1	7
Sun, 2014	1	0	1	0	1	0	1	4
Xu et al., 2014	1	0	1	0	1	1	1	5
Xu et al., 2015	1	1	1	1	1	1	1	7
Zhang et al., 2015	1	1	1	1	1	0	1	6
Duan et al., 2016	1	1	1	1	1	0	1	6
Mao et al., 2016	1	1	1	0	1	1	1	6
Wang et al., 2016	1	1	1	1	1	1	1	7
Zhu et al., 2016	1	0	1	0	1	1	1	5
Wang et al., 2017	1	0	1	0	1	1	1	5
Yang et al., 2017	1	0	1	0	1	0	1	4

A, adequate sequence generation; B, concealment of allocation; C, blinding of participants and personnel; D, blinding of outcome assessment; E, incomplete out-come data; F, selective reporting; G, other bias; 1, low risk of bias, the information of the domain was adequate in the text; 0, high risk of bias, the information of the domain was inadequate in the text.

### Effectiveness

#### The Incidence of MI and PCI

**Figure 2:**
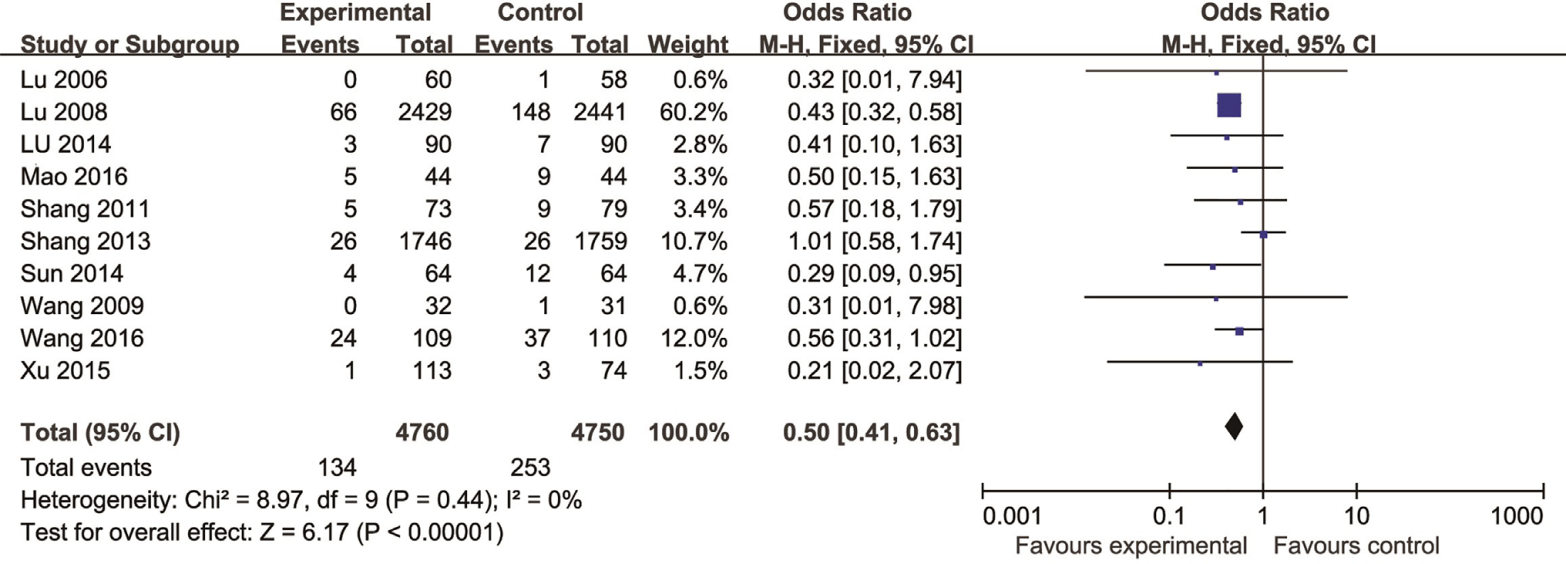
The forest plot: effects of Chinese herbal medicine for decreasing the incidence of myocardial infarction compared with control group.

**Figure 3:**
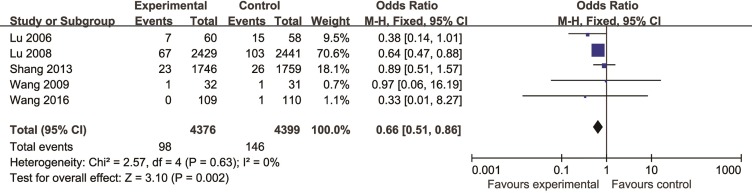
The forest plot: effects of Chinese herbal medicine for decreasing the incidence of percutaneous coronary intervention compared with control group.

Meta-analysis of 10 studies (Lu et al., 2006; Lu et al., 2008; Wang et al., 2009; Shang et al., 2011; Shang et al., 2013; Lu et al., 2014; Sun, 2014; Xu et al., 2015; Mao et al., 2016; Wang et al., 2016) found a significant difference in favor of CHM for decreasing the incidence of MI compared with control group (n = 9510, OR = 0.50, 95% CI (0.41, 0.63), P < 0.00001, I^2 =^ 0%) (**Figure 2**). Meta-analysis of five studies (Lu et al., 2006; Lu et al., 2008; Wang et al., 2009; Shang et al., 2013; Wang et al., 2016) showed CHM existed significant effect for decreasing the incidence of PCI compared with control group (n = 8775, OR = 0.66, 95% CI (0.51, 0.86), P = 0.002, I^2^ = 0%) (**Figure 3**).

### Cardiovascular Mortality

**Figure 4:**
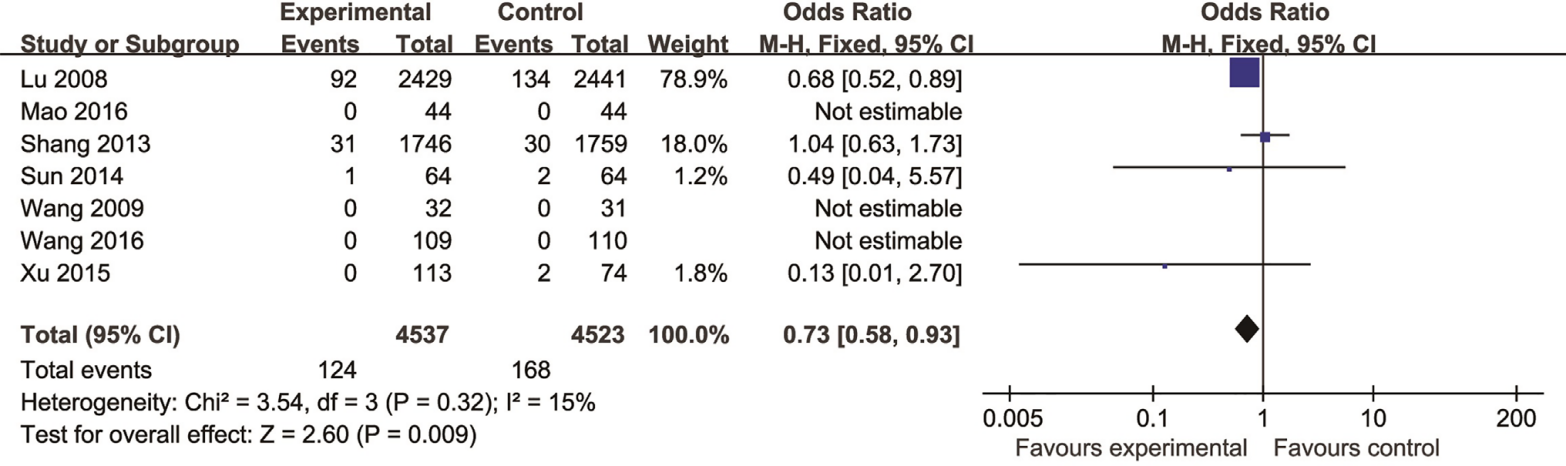
The forest plot: effects of Chinese herbal medicine for decreasing the cardiovascular mortality compared with control group.

Seven studies (Wang et al., 2009; Shang et al., 2013; Sun, 2014; Xu et al., 2015; Mao et al., 2016; Wang et al., 2016) reported cardiovascular mortality as the outcome measure. Of which, there were no deaths were found in three studies (Lu et al., 2008; Wang et al., 2009; Wang et al., 2016). Meta-analysis of remaining four studies (Shang et al., 2013; Sun, 2014; Xu et al., 2015; Mao et al., 2016) showed CHM existed significant effect for decreasing cardiovascular mortality compared with control group (n = 9,060, OR = 0.73, 95% CI: 0.58,0.93, P = 0.009, I^2^ = 15%) (**Figure 4**).

#### Systolic and Diastolic Functions of the Heart in Cardiac Ultrasound and the Level of ST-Segment Depression in Electrocardiogram

**Figure 5:**
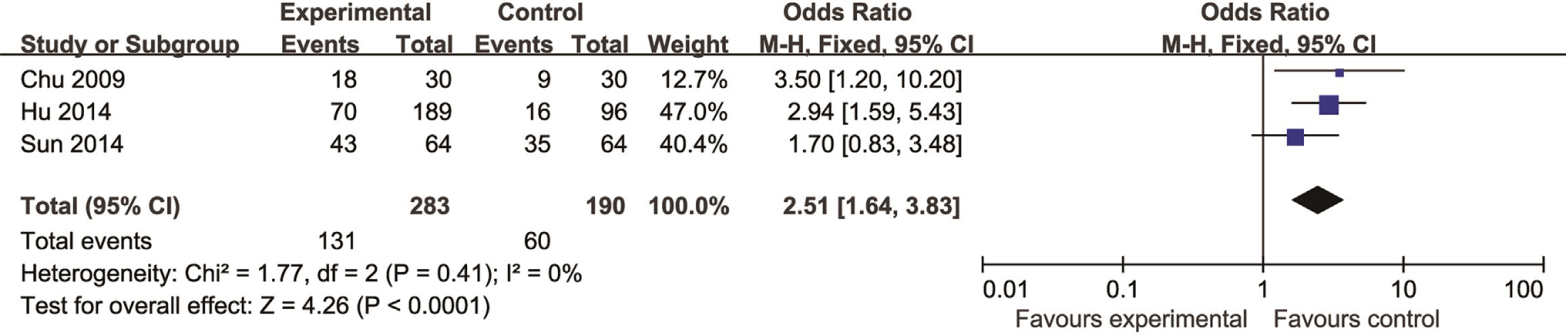
The forest plot: effects of Chinese herbal medicine for increasing degree of decline in the ST segment compared with control group.

For systolic function, five studies (Qiao et al., 2006; Qiu et al., 2009; Chen et al., 2013; Sun, 2014; Mao et al., 2016) showed CHM existed significant effect for increasing LVEF compared with control group (P < 0.05). For diastolic function, there was no study involving related indicators as outcome measure. Two studies (Qiao et al., 2006; Chen et al., 2013) showed that CHM could decrease the ventricular wall motion score compared with control (P < 0.05). In addition, meta-analysis of three studies (Chu et al., 2010; Hu et al., 2014; Sun, 2014) reported that CHM can increase degree of decline in the ST-segment compared with control (n = 473, OR = 2.51, 95% CI: 1.64∼3.83, P < 0.0001, I^2^ = 0%) (**Figure 5**).

#### Clinical Efficacy

**Figure 6:**
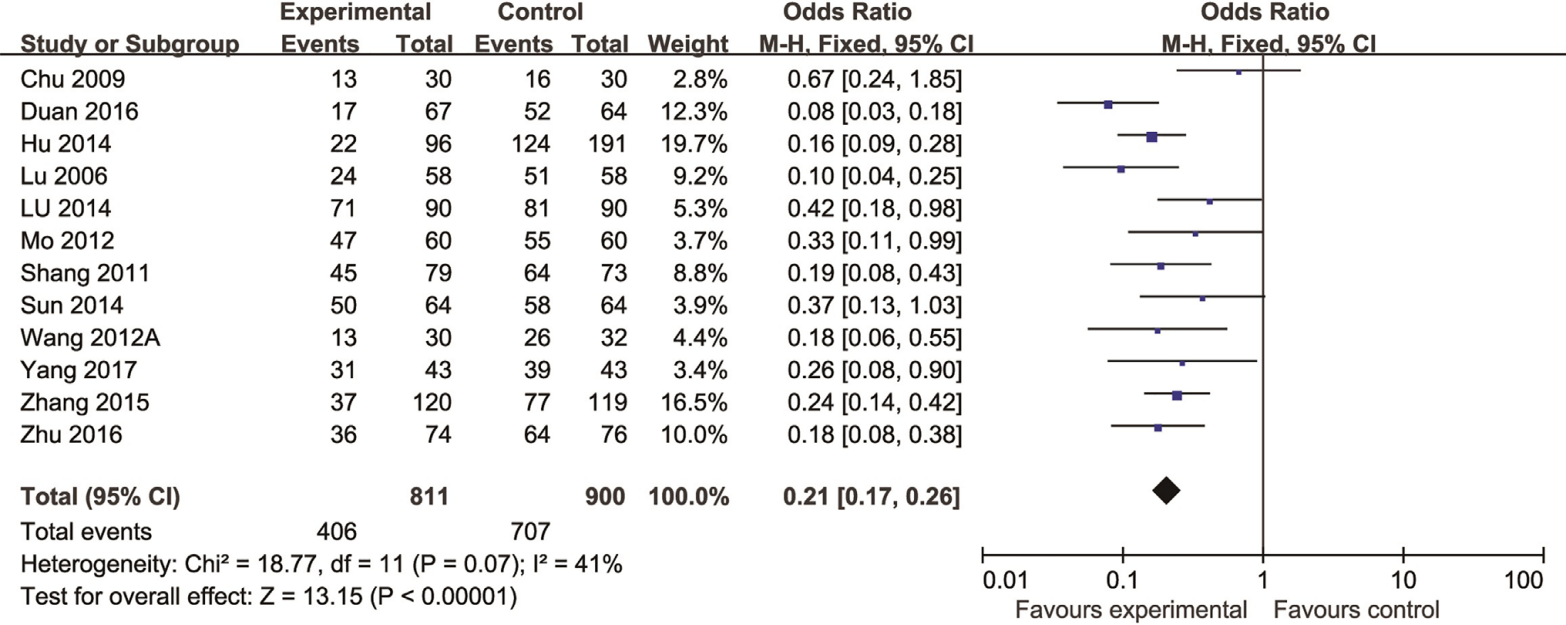
The forest plot: effects of Chinese herbal medicine for improving the efficiency of angina compared with control group.

**Figure 7:**
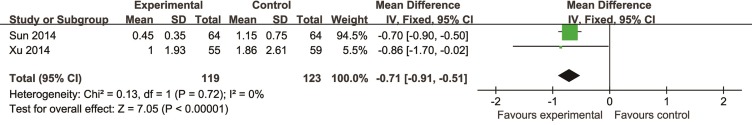
The forest plot: effects of Chinese herbal medicine for reducing the usage of nitroglycerin compared with control group.

**Figure 8:**
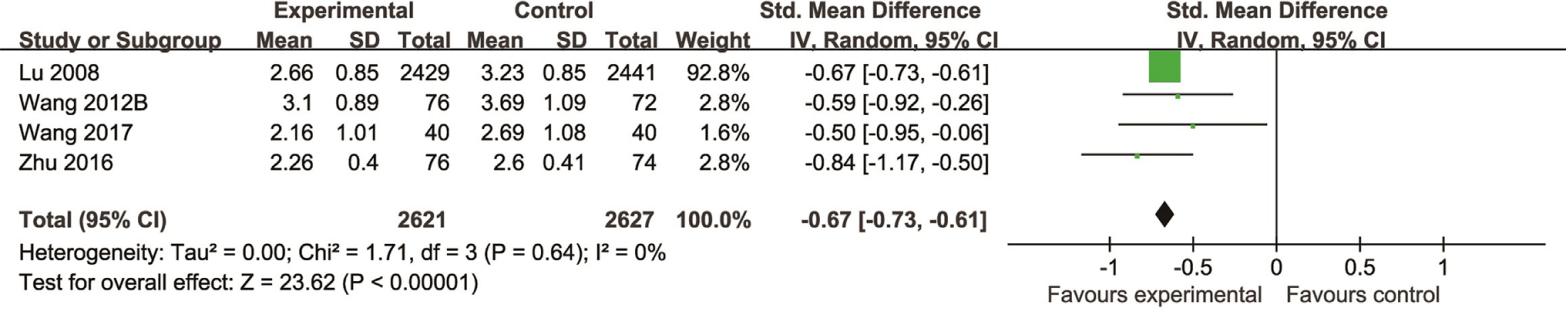
The forest plot: effects of Chinese herbal medicine for reducing low density lipoprotein.

**Figure 9:**
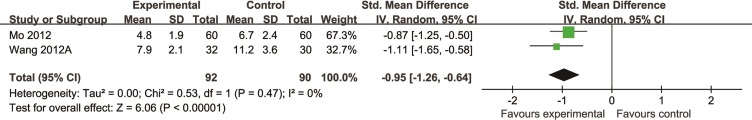
The forest plot: effects of Chinese herbal medicine for reducing hypersensitive C-reactive protein.

Compared with controls, meta-analysis of 12 studies (Lu et al., 2006; Chu et al., 2010; Shang et al., 2011; Mo et al., 2012; Wang S. H. et al., 2012; Hu et al., 2014; Lu et al., 2014; Sun, 2014; Zhang et al., 2015; Duan et al., 2016; Zhu et al., 2016; Yang et al., 2017) showed that the efficiency of angina improved more obviously in the TCM group than that in the control group (n = 1711, OR = 0.21, 95% CI: 0.17∼0.26, P = 0.09, I^2^ = 41%) (**Figure 6**); two studies (Sun, 2014; Xu et al., 2014) for reducing the usage of nitroglycerin (n = 242, MD = -0.71, 95%CI: -0.91∼-0.51, P < 0.00001, I^2^ = 0%) (**Figure 7**), four studies (Lu et al., 2008; Wang Y. G. et al., 2012; Zhu et al., 2016; Wang et al., 2017) for reducing LDL (n = 5,248, SMD = -0.67, 95%CI: -0.73∼-0.61, P < 0.00001,I^2^ = 0%) (**Figure 8**), two studies (Mo et al., 2012; Wang S. H. et al., 2012) for reducing hsCRP (n = 182, OR = -0.95, 95% CI: -1.26∼0.64, P < 0.00001, I^2^ = 0%) (**Figure 9**), two studies (Lu et al., 2006; Yang et al., 2017) for reducing the degree of coronary artery stenosis (P < 0.05), and two studies (Shang et al., 2011; Lu et al., 2014) for reducing the rate of coronary restenosis (P < 0.05).

### The Safety of Co-Administration of CHM

Adverse events were reported in 11 studies (Lu et al., 2006; Chu et al., 2010; Wang et al., 2009; Zhang et al., 2010; Shang et al., 2011; Shang et al., 2013; Sun, 2014; Zhang et al., 2015; Wang et al., 2016; Zhu et al., 2016; Yang et al., 2017), analyzed but not observed in four studies (Lu et al., 2008; Cheng et al., 2009; Chen et al., 2013; Liu et al., 2014), and not analyzed in 12 studies (Qiao et al., 2006; Qiu et al., 2009; Mo et al., 2012; Wang S. H. et al., 2012, Wang Y. G. et al., 2012; Lu et al., 2014; Hu et al., 2014; Xu et al., 2014; Xu et al., 2015; Duan et al., 2016; Mao et al., 2016; Wang et al., 2017). In the 11 studies with adequate information about adverse events, a total of 106/5,134 (2.06%) patients suffered adverse events in the treatment groups and 118/5,167 (2.28%) patients in control groups. Gastrointestinal discomfort symptoms, including nausea, stomachache, vomiting, diarrhea, anorexia, and constipation, were the most frequently occurring adverse events, affecting 74/106 (69.8%) patients in the treatment groups and 80/118 (67.8%) in control group patients. Allergy, hemorrhage, hepatic insufficiency, headache, and urinary tract infection were reported frequently also, affecting 20/106 (18.8%) patients in the treatment groups and 26/118 (22.0%) of patients in the control groups. The majority of above adverse events were mild and resolved by stopping related drugs and symptomatic treatment. Although some serious adverse events such as heart failure (1/106), cerebral hemorrhage (1/106), pericardial tamponade (1/106), coronary bypass surgery (1/106), and death (1/106) were reported in the two groups, there was no significant difference between the two groups.

### Ingredients of CHM Formulae and Frequently Used Herbs

The ingredients of CHM in each RCT are listed in **Table 3**. The most frequently used herbs across all formulae were *Miltiorrhiza* (nine formulae), pseudo-ginseng (seven formulae), ginseng (seven formulae), *Radix Paeoniae rubra* (six formulae), *Astragalus membranaceus* (five formulae), rhizome of *Chuanxiong* (five formulae), leech (five formulae), borneol (five formulae), and safflower (four formulae). Chinese *Angelica*, *Achyranthes bidentata*, *Rehmannia glutinosa*, peach kernel, liquorice, hawthorn, *Trichosanthes*, *Cinnamomum*, *Poria*, aloes, *Rhizoma Corydalis*, and ginkgo biloba were also frequently used.

**Table 3:** Ingredients of Chinese herbal medicine formulae.

Study (years)	Prescription	Ingredients of herb prescription	Usage of prescription	Preparations	Quality control
Chu et al., 2010	Xuefu Zhuyu capsule	Peach kernel, *Angelica sinensis*, rhizome of *Chuanxiong*, safflower, *Radix Paeoniae rubra*, *Radix Rehmanniae*, *Fructus aurantii*, *Radix Bupleuri*, *Platycodon grandiflorum*, *Radix Achyranthis bidentatae*, and liquorice	3#tid po	Capsule	Traditional Chinese patented medicine WY: Z12020223
Qiao et al., 2006 Chen et al., 2013	Tongguan capsule	*Astragalus membranaceus*, *Miltiorrhiza*, leech, etc.	3#tid po	Capsule	Produced by The Second Affiliated Hospital Of Guangzhou University Of Traditional Chinese Medicine
Cheng et al., 2009	Qingre Quyu granule	*Fructus trichosanthis* 15g, *Miltiorrhiza* 30g, hawthorn 30g, *Fritillaria thunbergii* 10g, pseudo-ginseng 3g, *Lignum Millettiae* 30g, and the seed of cowherb 15g	1#bid po	Decoction	Produced by China Pharmaceutical Materials Group Company
Lu et al., 2006 Shang et al., 2011	Xiongshao capsule	Rhizome of *Chuanxiong* and Radix *Paeoniae rubra*	2#tid po	Capsule	Unreported
Wang et al., 2017	Xuesaitong soft capsule	Pseudo-ginseng	2#bid po	Capsule	Traditional Chinese patented medicine WY: Z19990022
Mo et al., 2012	Yixin Mai granule	Ginseng, cassia twig, *Fructus trichosanthis*, leech, and *Poria cocos*	1#tid po	Decoction	Produced by Ruikang Hospital Affiliated to Guangxi College of Traditional Chinese Medicine
Liu et al., 2014	Red ginseng Tongxin granule	*Radix Paeoniae rubra* 10g, Agilawood 1g, *Angelica sinensis* 10g, orange peel 10g, *Rhizoma Corydalis* 6g, rhizome of *Chuanxiong* 6g, *Miltiorrhiza* 10g, astragalus 6g, peach kernel 10g, and safflower 10g	Unreported	Decoction	Produced by Jiangyin Tianjiang Pharmaceutical Co., Ltd.
Zhu et al., 2016	Traditional Chinese medicine prescription	Hawthorn, *Miltiorrhiza*, ginkgo leaf, lentil, *Psoralea*, sapanwood, *Ganoderma*, *Polygonum multiflorum*, *Cornus officinalis*, *Alisma orientalis*, *Radix Paeoniae alba*, cinnamon, *Pericarpium Citri reticulatae*, liquorice, *Fructus cnidii*, cicada slough, and *Ramuli Umcariae Cumuncis*	1#tid po	Capsule	Unreported
Wang et al., 2009	Shenshao tablet	Radix *Paeoniae alba* and ginseng	4#tid po	Tablet	Traditional Chinese patented medicine WY: Z19990059
Xu et al., 2015 Xu et al., 2014	Shenzhu Guanxin recipe	Ginseng 5g, *Rhizoma Atractylodis* 10g, *Radix Notoginseng* 10g, *Rhizoma Pinelliae* 10g, leech 3g, *Radix Panacis quinquefolium* 5g, and *Folium nelumbinis* 15g	50ml qd po	Decoction	Produced by Jiangxi Jiangyin Pharmaceutical Factory
Wang et al., 2012 Wang et al., 2016 Mao et al., 2016	Danlou tablet	*Fructus trichosanthis*, *Allium macrostemon*, the root of kudzu vine, rhizome of *Chuanxiong*, *Miltiorrhiza*, *Radix Paeoniae rubra*, *Alisma orientalis*, *Astragalus membranaceus*, *Curcuma aromatica*, and *Drynaria* rhizome	4.5g qd po	Tablet	Traditional Chinese patented medicine WY: YBZ17382006
Zhang et al., 2010 Lu et al., 2014	Tongxinluo capsule	Ginseng, leech, scorpion, red peony root, cicada slough, soil turtle worm, centipede, sandalwood, *Lignum acronychiae*, frankincense, jujube nut, and borneol	Before PCI: 8# qd po After PCI: 4#tid po	Capsule	Traditional Chinese patented medicine WY: Z19980015
Qiu et al., 2009	Compound *Salvia* tablet and Xinyue capsule	*Miltiorrhiza* 450mg, pseudo-ginseng 141mg, borneol 8mg, ginseng 50mg	Unreported	Capsule	Traditional Chinese patented medicine WY: Z44023372 and Z20030073
Hu et al., 2014	Kodaling tablet	Rhizoma *Corydalis*	3#tid po	Tablet	Produced by Zhejiang KangEnbei Pharmaceutical Co., Ltd.
Duan et al., 2016	Live heart pill	Ginseng, *Radix Aconiti carmichaeli*, *Ganoderma lucidum*, safflower, musk, bezoar, bear bile, pearl, toad venom, and borneol	2#tid po	Tablet	Traditional Chinese patented medicine WY: Z44021835
Zhang et al., 2015	Wufuxinnaoqing	Safflower oil, borneol, vitamin E, and vitamin B6	2#tid po	Capsule	Produced by Shineway Pharmaceutical Group Co., Ltd.
Lu et al., 2008	Xuezhikang capsule	Red yeast Chinese rice	600mg bid po	Capsule	Produced by the Beijing WBL Peking University Biotech Co. Ltd.
Shang et al., 2013	Qi-Shen-Yi-Qi dripping pills	*Miltiorrhizae*, pseudo-ginseng, *Lignum Dalbergiae odoriferae*, and *Astragalus membranaceus*	0.5g tid po	Pill	Unreported in detail
Yang et al., 2017	Coronary Ningtong prescription	*Astragalus membranaceus* 30g, *Miltiorrhiza* 30g, mulberry parasitism 30g, *Gynostemma pentecox* 30g, hawthorn 30g, the root of kudzu vine 30g, Herba *Rhodiolae* 30g, *Fructus trichosanthis* 15g, *Allium macrostemon* 15g, *Rhizoma Pinellinae praeparata* 15g, immature bitter orange 10g, safflower 10g, *Rhizoma Sparganii* 10g, *Zedoaria* 10g, *Rhizoma coptidis* 6g, pseudo-ginseng 3g, and leech 3g	100ml bid po	Decoction	Unreported
Sun, 2014	Musk Baoxin pill	artificial musk, ginseng, cinnamon, toad venom, storax, artificial bezoar, and borneol	2#tid po	Pill	Produced by Shanghai and Huangyao Pharmaceutical Industry
Wang et al., 2012	Double ginseng capsule And Tongguan capsule	*Miltiorrhiza*, ginseng, *Herba Rhodiolae*, pseudo-ginseng and *Lignum Dalbergiae odoriferae*	4#tid po	Capsule	Produced by Shaanxi Pharmaceutical Group Shaanxi New Drug Technology Development Center

d, day; #: tablet; PCI, Percutaneous Coronary Intervention; Co., Ltd, Company Limited.

### Possible Mechanism of Herbal Benefits for CHD

A total of 45 experimental studies (Jormalainen et al., 2004; Liu et al., 2008; Chen et al., 2008; He et al., 2008; Nizamutdinova et al., 2008; Wang et al., 2008; Liu et al., 2010; Liu et al., 2010; Tang et al., 2010; Zhang et al., 2010; Liu and Niu, 2011; Liu et al., 2011; Pan et al., 2011; Wu et al., 2011; Zhai et al., 2011; Liu et al., 2012; Lv et al., 2012; Zhu et al., 2013; Lim et al., 2013; Tu et al., 2013; Wang et al., 2013; Yin et al., 2013; Zhang et al., 2013; He et al., 2014; Li et al., 2014; Liu et al., 2014; Park et al., 2014; Qian et al., 2014; Tang et al., 2014; Tao et al., 2014; Wei et al., 2014; Xue et al., 2014; Zhang et al., 2014; Deng et al., 2015; Lu et al., 2015; Wang et al., 2015; Xia et al., 2015; Yu et al., 2015; Chen et al., 2016; Fan et al., 2016; Hu et al., 2016; Leng et al., 2015; Ma et al., 2016; Meng et al., 2016; Yu et al., 2016) were identified in our electronic searches to investigate the effects and mechanisms of the main active components of single flavored Chinese medicine which were frequently used on I/R injury models (**Table 4**). The possible mechanisms of them are summarized as follows: (1) oxidative stress is important reaction after myocardial ischemia. The function of free radical scavenging system is decreased in myocardial ischemia. Large amounts of free radicals was produced by the unbalanced endogenous antioxidant systems, which further leads to the peroxidation of lipids, proteins and nucleic acids, the biochemical alteration (reducing SOD, and GSH-Px, and increasing MDA), and further led to cardiomyocyte death (Yellon and Hausenloy, 2007). Based on these observations, antioxidant therapy is the key step considered to prevent I/R injury. In our study, *Salvia miltiorrhiza*, salvianolic acid B, tanshinone IIA, notoginsenoside R1, ginsenoside Rb1, ginsenoside Rb3, astragaloside IV, and ligustrazine could enhance SOD (Chan et al., 2012; Lv et al., 2012; Liu et al., 2014; Tang et al., 2014; Xue et al., 2014; Wang et al., 2015; Xia et al., 2015) and attenuate chondriokinesis to reduce the release of MDA (Liu et al., 2011; Chan et al., 2012; Liu et al., 2014; Tang et al., 2014; Xue et al., 2014; Xia et al., 2015); borneol, ginsenoside Rd, and hydroxysafflor yellow A could reduce ROS (He et al., 2008; Liu and Niu, 2011; Wang et al., 2013). *S. miltiorrhiza* and hydroxysafflor yellow A (Hu et al., 2016) exhibit antioxidant effects *via* PI3K/Akt signaling pathway; tanshinone IIA (Wei et al., 2014) increases NADPH oxidase *via* AMPK/Akt/PKC pathway; and astragaloside IV (Zhang et al., 2014) could reduce ROS *via* the PI3K/Akt/mTOR pathway. Our study showed TCM could improve the antioxidant function to reduce the damage of myocardial ischemia. (2) Apoptosis was an energy-requiring programmed cell death (Zhang and Xu, 2000). Apoptosis can be activated extrinsically by sarcolemmal receptors such as FAS: FAS(CD 95) and tumor necrosis factor alpha (TNF-α) (Kleinbongard et al., 2011), or intrinsically by cytochrome c which initiates the caspase cascade activation result in intracellular proteolysis. In addition, the opening of mitochondrial permeability transition pore (MPTP) conduces the mitochondrial matrix swelling, then leading to rupture of the outer membrane and release of cytochrome c, activating the caspase cascade, ultimately resulting in the apoptotic cell death (Heusch et al., 2010). Proapoptotic and antiapoptotic proteins of the Bcl family interact with the MPTP (Baines, 2009). In present study, *S. miltiorrhiza*, salvianolic acid A, salvianolic acid B, paeonol, paeoniflorin, ginsenoside Rb1, ginsenoside Rb3, ginsenoside Rd, ginsenoside Rg3, and ligustrazine could increase Bcl-2 expression (Nizamutdinova et al., 2008; Wang et al., 2008; Tang et al., 2010; Zhai et al., 2011; Wang et al., 2013; Liu et al., 2014; Wang et al., 2015; Fan et al., 2016) and the Bcl-2/Bax ratio (Nizamutdinova et al., 2008; Tang et al., 2010; Wu et al., 2011; Zhai et al., 2011; Wang et al., 2013; Liu et al., 2014; Wang et al., 2015; Chen et al., 2016; Fan et al., 2016). Three studies (Wang et al., 2008; Zhang et al., 2013; Chen et al., 2016) reported that salvianolic acid A, tanshinone IIA, and ginsenoside Rb1 exhibit anti-apoptotic effects *via* PI3K/Akt signaling pathway, and one study (Wang et al., 2013) reported that ginsenoside Rd could decrease caspase-3 and caspase-9 activities. (3) The inflammation during myocardial I/R injury was reviewed by previous studies (Marchant et al., 2012). The excessive inflammation can lead to cardiomyocyte damage. When the myocardium got reperfused, the NF-κB pathway was activated by pattern recognition receptors, culminating in promoted cytokine expression. *S. miltiorrhiza*, tanshinone I, tanshinone IIA, paeonol, notoginsenoside r1, ginsenoside Re, ginsenoside Rg1, ligustrazine, astragaloside IV, and astragalus polysaccharides were shown to exert anti-inflammatory effects by decreasing TNF-alpha (Zhang et al., 2010; Lim et al., 2013; Tu et al., 2013; Deng et al., 2015; Lu et al., 2015; Xia et al., 2015), IL-6 (Chen et al., 2008; Zhang et al., 2010), IL-8 (Li et al., 2014), and NF-κB (Tu et al., 2013; Qian et al., 2014; Deng et al., 2015; Lu et al., 2015). Two studies (Qian et al., 2014; Zhu et al., 2013) reported that ligustrazine and astragalus polysaccharides exhibit anti-inflammatory effects *via* inhibiting P38MAPK pathway, and one study (Zhang et al., 2010) reported that tanshinone IIA could decrease TNF-alpha and IL-6 *via* PI3K/Akt pathway. (4) Nitric oxide is an essential modulator of cardiovascular system. The NO can decrease intracellular calcium concentration in vascular smooth muscle cells, which further induces vasodilation (Schulz et al., 2004). Salvianolic acid B, tanshinone IIA, ginsenoside Rb1, ginsenoside Rg3, ligustrazine, astragaloside IV, and hydroxysafflor yellow A were shown to improve circulation by increasing NO expression (Liu et al., 2008; Liu et al., 2010; Pan et al., 2011; Lv et al., 2012; Leng et al., 2015; Wang et al., 2015) *via* up-regulating eNOS phosphorylation (Liu et al., 2008; Liu et al., 2010; Pan et al., 2011; Lv et al., 2012; Leng et al., 2015; Wang et al., 2015). (5) *S. miltiorrhiza* and notoginsenoside r1 were shown to regulate energy metabolism *via* p-JNK-NF-kappaB-TRPC6 pathway (Meng et al., 2016) and ROCK-dependent ATP5D modulation separately (He et al., 2014; Li et al., 2014). (6) Hirudin was shown to attenuate coagulation and enhance microvascular flow during reperfusion (Jormalainen et al., 2004). Thus, antioxidant, anti-apoptotic, circulation improvement, anti-inflammatory, and energy metabolism regulation actions have been promoted as important mechanisms of herbal compounds used to treat I/R injury.

**Table 4:** Mechanisms of the main active components of single flavored Chinese Medicine on organic injury induced by ischemia/reperfusion.

Active ingredients	Herb source	Possible mechanisms (signaling pathway)	Citation	Structure
Salvia miltiorrhiza	Miltiorrhiza	1. Regulation of energy metabolism (p-JNK-NF-kappaB-TRPC6 pathway) 2. Attenuation of oxidative stress (Akt/Nrf2/HO-1 pathway) 3. Anti-inflammation 4. Anti-apoptosis (increase expression of Bcl-2 and increase Bcl-2/Bax ratio, affect Akt, and ERK1/2 phosphorylation)	1. Meng et al., 2016 2. Hu et al., 2016 3. Yin et al., 2013 4. Yu et al., 2015; Fan et al., 2016	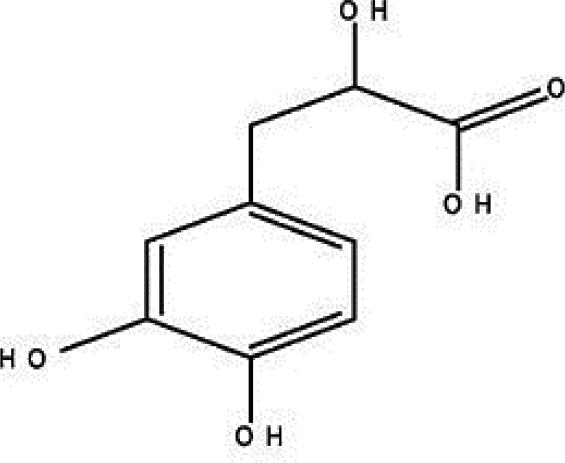
Salvianolic acid A	Miltiorrhiza	1. Anti-apoptosis (increase Bcl-2/Bax ratio *via* JNK/PI3K/Akt signaling pathway)	1. Chen et al., 2016	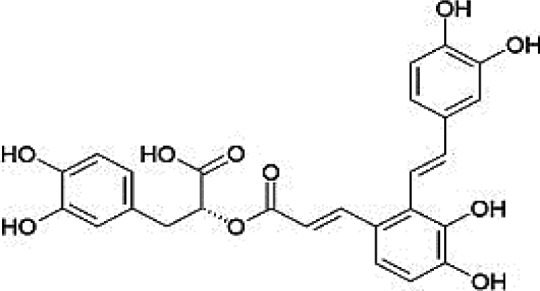
Salvianolic acid B	Miltiorrhiza	1. Improve circulation (increase expression of NO *via* up-regulating eNOS phosphorylation) 2. Attenuation of oxidative stress (increase SOD and decrease MDA) 3. Anti-apoptosis	1. Pan et al., 2011 2. Xue et al., 2014 3. Xue et al., 2014	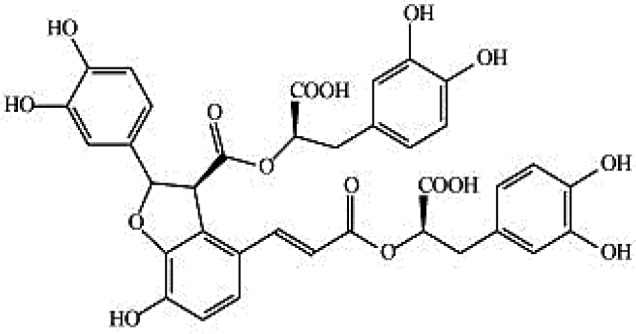
Tanshinone I	Miltiorrhiza	1. Anti-inflammation	1. Park et al., 2014	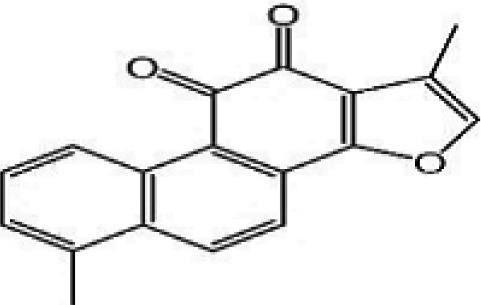
Tanshinone IIA	Miltiorrhiza	1. Improve circulation (increase expression of NO *via* up-regulating eNOS phosphorylation) 2. Attenuation of oxidative stress (increase SOD and HO-1, decrease MDA, increase NADPH oxidase *via* AMPK/Akt/PKC pathway) 3. Anti-inflammation (decrease TNF-alpha and IL-6 *via* PI3K/Akt-dependent pathway) 4. Anti-apoptosis (decrease caspase-3 activity *via* Akt/FOXO3A/Bim-mediated signal pathway)	1. Pan et al., 2011 2. Tang et al., 2014; Wei et al., 2014 3. Zhang et al., 2010 4. Zhang et al., 2013	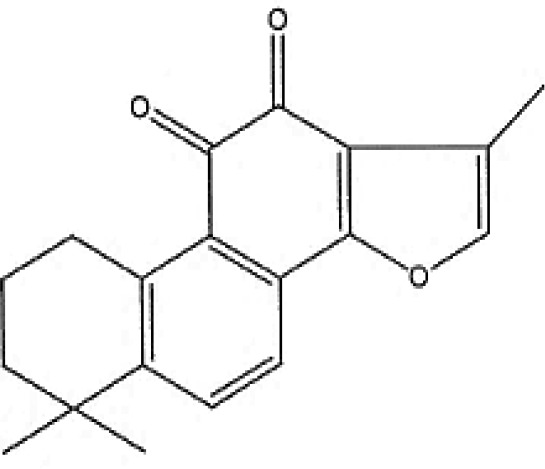
Paeonol	Radix Paeoniae rubra	1. Anti-inflammation 2. Anti-apoptosis (increase expression of Bcl-2 and increase Bcl-2/Bax ratio)	1. Ma et al., 2016 2. Nizamutdinova et al., 2008	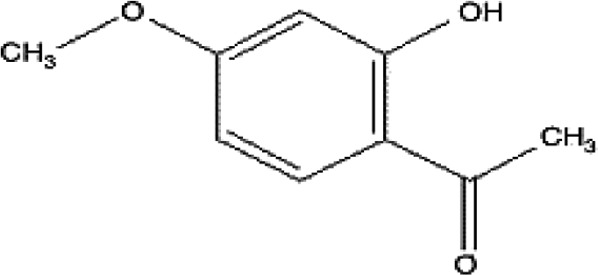
Paeoniflorin	*Radix Paeoniae rubra*	1. Anti-apoptosis (increase expression of Bcl-2 and increased Bcl-2/Bax ratio)	1. Tang et al., 2010	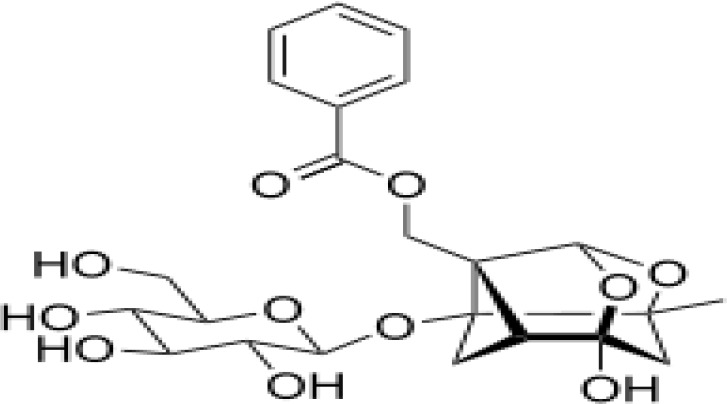
Notoginsenoside r1	Pseudo-Ginseng	1. Anti-inflammation (decrease IL-6, IL-8, and TNF-alpha) 2. Regulation of energy metabolism (ROCK-dependent ATP5D modulation) 3. Anti-apoptosis (increase Bcl-2 expression) 4. Attenuation of oxidative stress (increase SOD, and decrease MDA) 5. Attenuation of endoplasmic reticulum stress	1. Chen et al., 2008; Li et al., 2014; Xia et al., 2015 2. He et al., 2014; Li et al., 2014 3. Liu et al., 2010 4. Xia et al., 2015 5. Yu et al., 2016	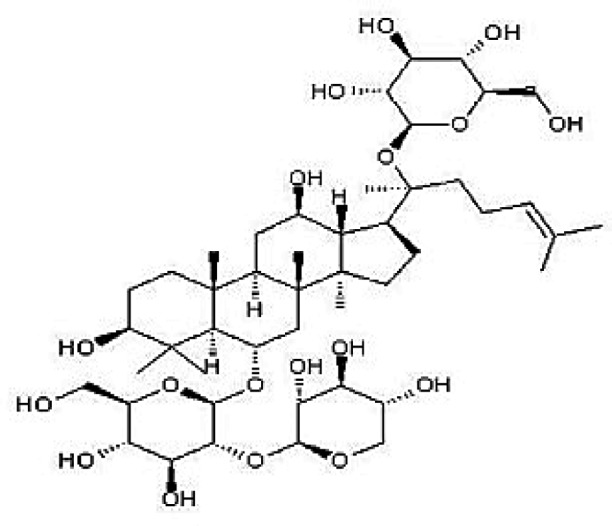
Ginsenoside Rb1	Ginseng	1. Anti-apoptosis (increase Bcl-2 expression and increased Bcl-2/Bax ratio *via* PI3K/Akt pathway) 2. Attenuation of oxidative stress (increase SOD, and decrease MDA) 3. Improve circulation (increase NO expression *via* up-regulating eNOS phosphorylation)	1. Wang et al., 2008; Wu et al., 2011 2. Chan et al., 2012 3. Leng et al., 2015	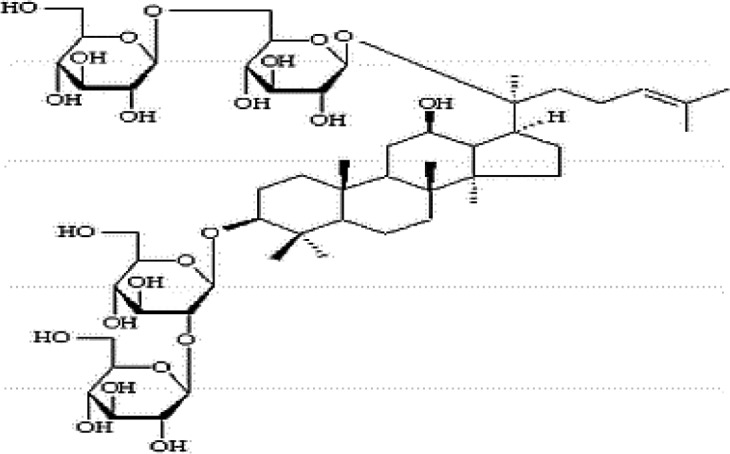
Ginsenoside Rb3	Ginseng	1. Attenuation of oxidative stress (increase SOD, and decrease MDA) 2. Anti-apoptosis (increase Bcl-2 expression and increase Bcl-2/Bax ratio)	1. Liu et al., 2014 2. Liu et al., 2014	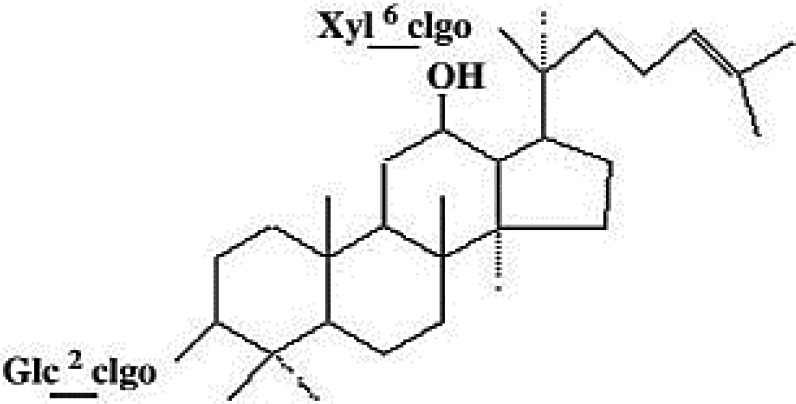
Ginsenoside Rd	Ginseng	1. Attenuation of oxidative stress (decrease ROS) 2. Anti-apoptosis (increase Bcl-2 expression and increase Bcl-2/Bax ratio, decrease caspase-3 and caspase-9 activity *via* mitochondrial-dependent apoptotic pathway)	1. Wang et al., 2013 2. Wang et al., 2013	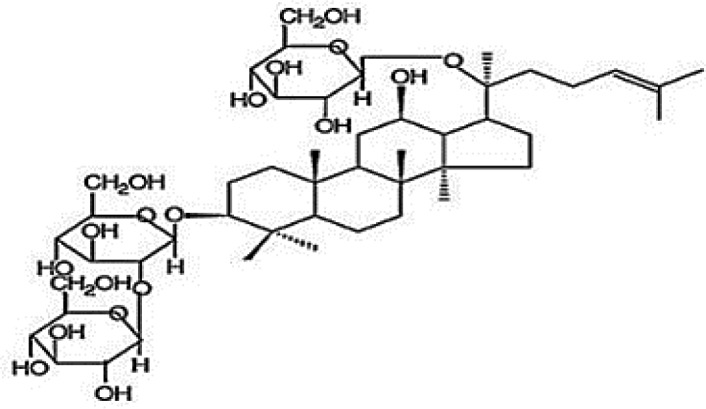
Ginsenoside Re	Ginseng	1. Anti-inflammation (decrease TNF-alpha)	1. Lim et al., 2013	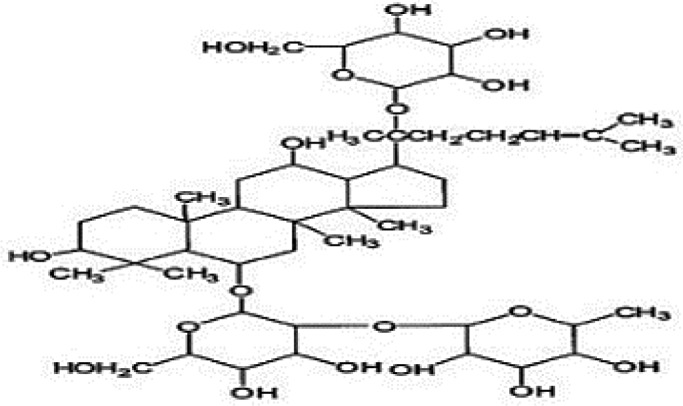
Ginsenoside Rg1	Ginseng	1. Anti-inflammation (decrease TNF-alpha and IL-1beta, in part *via* the NF-κB signaling pathway)	1. Tao et al., 2014; Deng et al., 2015	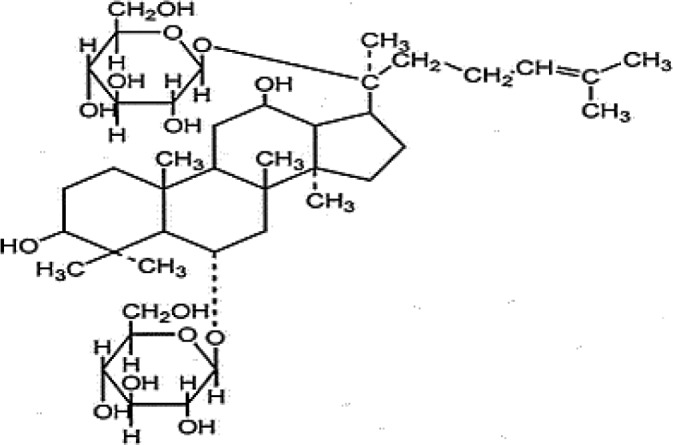
Ginsenoside Rg3	Ginseng	1. Attenuation of oxidative stress (increase SOD) 2. Anti-apoptosis (increase Bcl-2 expression and increase Bcl-2/Bax ratio) 3. Improve circulation (increase NO expression *via* up-regulating eNOS phosphorylation)	1. Wang et al., 2015 2. Wang et al., 2015 3. Wang et al., 2015	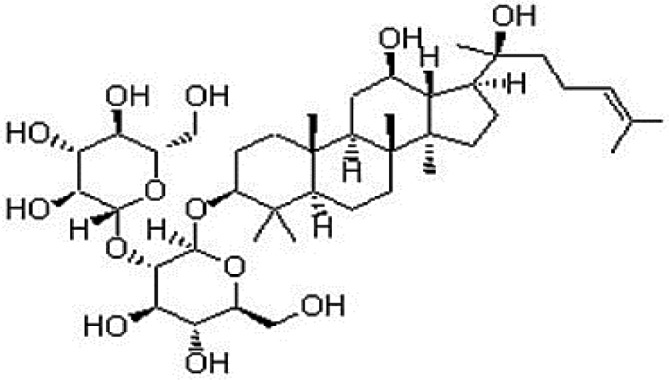
Borneol	Borneol	1. Attenuation of oxidative stress (decrease ROS)	1. Liu and Niu, 2011	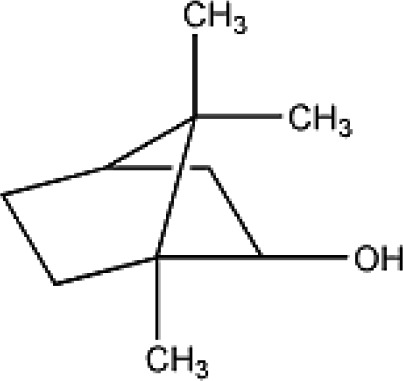
Ligustrazine	Rhizome of *Chuanxiong*	1. Attenuation of oxidative stress (increase SOD and decrease MDA) 2. Improve circulation (increase NO expression *via* up-regulating eNOS phosphorylation) 3. Anti-inflammation (decrease the expression of NF-κB *via* inhibiting P38MAPK pathway) 4. Anti-apoptosis (increase Bcl-2 expression and increase Bcl-2/Bax ratio)	1. Liu et al., 2011; Lv et al., 2012 2. Lv et al., 2012 3. Qian et al., 2014 4. Zhai et al., 2011	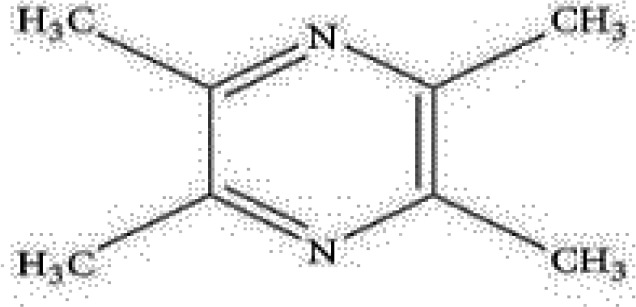
Astragaloside IV	Astragalus membranaceus	1. Promoting angiogenesis (increase the expression of VEGF) 2. Anti-apoptosis (increase Bcl-2 expression and increase Bcl-2/Bax ratio, decrease caspase-3) 3. Anti-inflammation (decrease the expression of TNF-alpha and NF-κB) 4. Improve circulation (increase NO expression *via* up-regulating eNOS phosphorylation) 5. Attenuation of oxidative stress (reduce ROS to *via* the PI3K/Akt/mTOR pathway)	1. Yu et al., 2015 2. Tu et al., 2013; Lu et al., 2015 3. Tu et al., 2013; Lu et al., 2015 4. Liu et al., 2010 5. Zhang et al., 2014	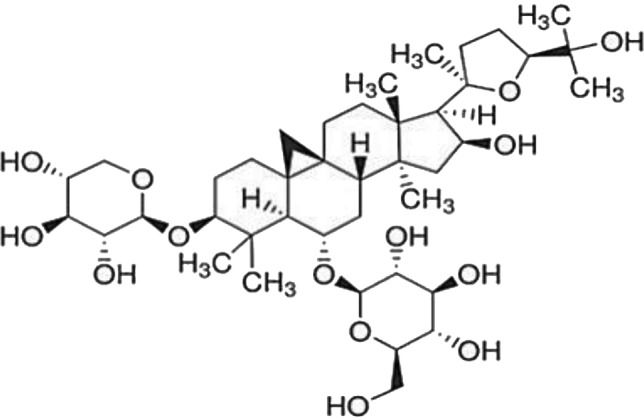
Astragalus polysaccharides	Astragalus membranaceus	1. Anti-inflammatory (*via* the p38 MAPK signaling pathway)	1. Zhu et al., 2013	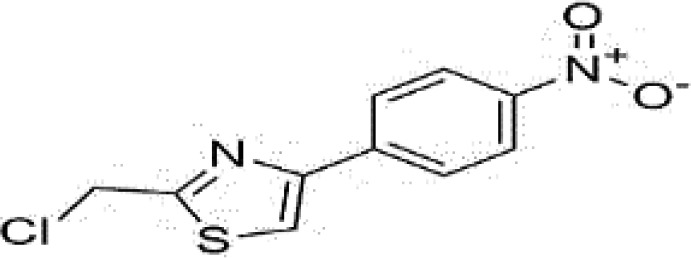
Hirudin	Leech	1. Attenuate coagulation and enhance microvascular flow during reperfusion	1. Jormalainen et al., 2004	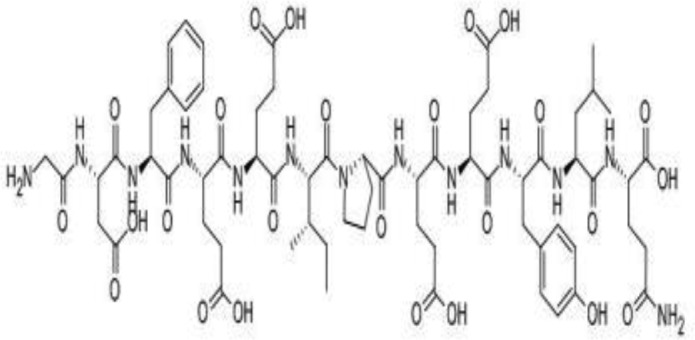
Hydroxysafflor yellow A	Safflower	1. Attenuation of oxidative stress (Akt/Nrf2/HO-1 pathway, decrease ROS) 2. Improve circulation (increase NO expression *via* up-regulating eNOS phosphorylation)	1. He et al., 2008; Hu et al., 2016 2. Liu et al., 2008	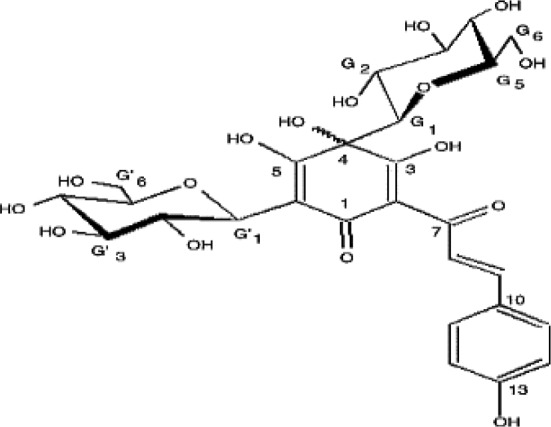

HO-1, heme oxygenase-1; SOD, super oxide dismutase; PI3K, phosphatidylinositol 3-kinase; ROS, reactive oxygen species; eNOS, endothelial nitric oxide synthase; MDA, malonaldehyde; NADPH, nicotinamide adenine dinucleotide phosphate; VEGF, vascular endothelial growth factor; NF-κB, nuclear factor κB.

## Discussion

### Summary of Evidence

This is the first clinical systematic review of 27 high-quality RCTs involving 11,732 participants to estimate the efficacy and safety of CHMs for CHD. The evidence available from present study revealed that CHMs are beneficial for CHD and are generally safe. In addition, CHM exerted cardioprotection for CHD, possibly altering multiple signal pathways through anti-inflammation, anti-oxidation, anti-apoptosis, circulation improvement, and energy metabolism regulation.

### Limitations

First, there were still some methodological weaknesses in the primary studies although we included high-quality studies. Only nine of the 27 included studies (Lu et al., 2006; Chu et al., 2010; Wang et al., 2009; Liu et al., 2014; Xu et al., 2015; Zhang et al., 2015; Duan et al., 2016; Mao et al., 2016; Wang et al., 2016) reported allocation concealment, and eight included studies (Lu et al., 2006; Chu et al., 2010; Wang et al., 2009; Liu et al., 2014; Xu et al., 2015; Zhang et al., 2015; Duan et al., 2016; Wang et al., 2016) reported blinding during outcome assessment. It is worth noting that an average 18% more “beneficial” effect in trials with inadequate or unclear concealment of allocation compared with adequate concealment (Higgins and Green, 2012). And blinding during outcome assessment is an essential method to avoid systemic errors which existed in the outcome assessment of non-blinded studies (Higgins and Green, 2012). Second, English and Chinese literatures were included only in present study and the absence of studies written in other languages may generate selective bias in a certain degree. Third, no included trials were reported to have been registered, and negative findings were less likely to be published, which may lead to the efficacy being overestimated.

### Implications

The findings from present study indicate that CHM paratherapy is beneficial for CHD and is well tolerated. Thus, we recommended, at least to an extent, to use CHMs for CHD, especially selected case. Further study should identify specific CHM and/or indications of CHM. In addition, the findings of the most frequently used herbs such as *Miltiorrhiza*, pseudo-ginseng, ginseng, *Radix Paeoniae rubra*, *Astragalus membranaceus*, rhizome of *Chuanxiong*, leech, borneol, and safflower and their main active components should be considered as further development of herbal prescriptions and component injection for CHD.

Some methodological weaknesses still existed in the primary studies. Recommendations for further research are as follows: (1) the CONSORT 2010 statement (Schulz et al., 2010), CONSORT for TCM (Bian et al., 2011), RCTs investigating CHM (Flower et al., 2012), and CONSORT Extension for Chinese Herbal Medicine Formulas 2017 (Cheng et al., 2017) should be abided by for the design. (2) Clinic trials should be registered in a generally accessible database (www.clinicaltrials.com) prior to first case inclusion. It allows verification of predefined study hypothesis and end-points of the study, which would help to the report of negative findings and reduce publication bias (Rongen and Wever, 2015). (3) In view of trials with insufficient statistical power that runs the risk of over estimating therapeutic efficacy (Kjaergard et al., 2001), the further studies are recommended to provide statistical information of sample size estimation. (4) In order to ensure the efficacy of TCM, the identity and quantity of the herbal preparations should be described clearly in further research. (5) The safety of TCM has been increasingly concerned by both medical workers and the public.

The frequency of use for particular herb was calculated and those used at a high frequency that are described in detail in the part 3.6 and **Table 3**. The high-frequency herbs that we selected can ignite *the treatment based on syndrome differentiation* according to the herbal functions **Table 5**. Ginseng and *Astragalus membranaceus* benefit qi; *Miltiorrhiza*, pseudo-ginseng, *Radix Paeoniae rubra*, rhizome of *Chuanxiong*, leech, and safflower promote blood circulation for removing blood stasis; and borneol has function of resuscitation with aromatics for relieving pain. Thus, we can also deduce that the main patterns of CHD are qi deficiency and blood stasis. The selected high-frequency herbs are composed of a herbal prescription for CHD, which can be used for clinic and as a candidate for RCT.

**Table 5:** Different syndromes of coronary heart disease and the classification of herbs according to syndrome differentiation therapy for different syndromes.

Syndrome	Syndrome differentiation therapy for different syndromes	Representative herbs in the theory of traditional Chinese medicine mentioned in present study
Syndrome of coagulation cold in heart vessel	1. Dispelling cold 2. Dredging channel blockade and yang	1. Cinnamon, *Psoralea*, *Ramuli Umcariae Cumuncis* 2. *Angelica sinensis*, radix *Paeoniae rubra*, pseudo-ginseng, rhizome Of *Chuanxiong*, frankincense, *Miltiorrhiza*, safflower, peach kernel, rhizoma *Corydalis*, leech, soil turtle worm, *Lignum Millettiae*, ginkgo leaf, sapanwood, red yeast Chinese, *Allium macrostemon*
Syndrome of qi stagnation in heart and chest	1. Dispersing stagnated liver qi for regulating qi-flowing	1. Hawthorn, *Fructus aurantii*, *Lignum acronychiae*, Agilawood, radix *Bupleuri*, rhizoma *Corydalis*
Syndrome of blockade of heart blood	1. Promoting blood circulation for removing blood stasis	1. *Angelica sinensis*, radix *Paeoniae rubra*, pseudo-ginseng, rhizome Of *Chuanxiong*, frankincense, *Miltiorrhiza*, safflower, peach kernel, rhizoma *Corydalis*, leech, soil turtle worm, *Lignum Millettiae*, ginkgo leaf, sapanwood, red yeast Chinese rice
Syndrome of turbid phlegm blocking heart	1. Dredging yang for resolving turbidity 2. Eliminating phlegm for resolving masses	1. *Allium macrostemon* 2. *Rhizoma Pinelliae*, liquorice, *Platycodon grandiflorum*, *Fritillaria thunbergii*, orange peel
Syndrome of deficiency of both qi and yin	1. Benefiting qi and nourishing yin 2. Promoting blood circulation for dredging vessels	1. *Angelica sinensis*, ginseng, *Astragalus Membranaceus*, radix *Panacis quinquefolium*, *Rhizoma Atractylodis*, liquorice 2. *Radix Paeoniae Rubra*, pseudo-ginseng, rhizome Of *Chuanxiong*, frankincense, *Miltiorrhiza*, safflower, peach kernel, rhizoma *Corydalis*, leech, soil turtle worm, *Lignum Millettiae*, ginkgo leaf, sapanwood, red yeast Chinese rice, musk
Syndrome of yin deficiency of heart and kidney	1. Nourishing yin and clearing heat 2. Activating blood circulation for nourishing heart	1. *Angelica sinensis*, borneol, *Cassia* twig, cicada slough, *Fructus trichosanthis*, *Fritillaria thunbergii*, orange peel, *Folium nelumbinis*, bezoar, bear bile, pearl, toad venom, *Gynostemma pentecox*, *Rhizoma Coptidis*, liquorice 2. *Radix Paeoniae rubra*, pseudo-ginseng, rhizome of *Chuanxiong*, frankincense, *Miltiorrhiza*, safflower, peach kernel, rhizoma *Corydalis*, leech, soil turtle worm, *Lignum Millettiae*, ginkgo leaf, sapanwood, red yeast Chinese rice
Syndrome of yang deficiency of heart and kidney	1. Warmly tonifying yang qi and inspiring heart yang	1. Ginseng, *Astragalus Membranaceus*, radix *Panacis quinquefolium, Rhizoma Atractylodis*, liquorice, *Ganoderma*, *Herba Rhodiolae*

Cardioprotection by anti-inflammation, antioxidant, anti-apoptosis, and circulation improvement for myocardial I/R injury (Xu et al., 2014) was an innovative strategy for antagonizing the injurious biochemical and molecular events that eventually resulted in irreversible ischemic injury (Wu and He, 2010). The included preclinical trials presented the main active components of the most frequently used herbs that performed anti-inflammatory, anti-oxidation, anti-apoptosis, energy metabolism regulation, and circulation improvement mechanisms in multiple models of I/R injury through multiple signal pathways, including the PI3K/Akt signaling pathway, AMPK/Akt/PKC pathway, PI3K/Akt/mTOR pathway, mitochondrial-dependent apoptotic pathway, P38MAPK pathway, eNOS phosphorylation, and p-JNK-NF-kappaB-TRPC6 pathway. Further studies of CHM for CHD should explore the multi-drug, multi-target signal pathway using novel techniques such as network pharmacological approach.

### Conclusion

The findings from present study indicate that CHMs are beneficial for CHD and are generally safe. In addition, CHM exerted cardioprotection for CHD, possibly altering multiple signal pathways through anti-inflammatory, anti-oxidation, anti-apoptosis, circulation improvement, and energy metabolism regulation mechanisms.

## Author Contributions

Study conception and design: GQZ, YW, KJZ, and QZ. Acquisition, analysis and/or interpretation of data: QZ, KJZ, JZZ, XYB, QT, PCZ, ZZ, YYH, GQZ, YW. Final approval and overall responsibility for this published work: GQZ and YW.

## Funding

This project was supported by the grant of National Natural Science Foundation of China (81573750/81473491/81173395/H2902).

## Conflict of Interest Statement

The authors declare that the research was conducted in the absence of any commercial or financial relationships that could be construed as a potential conflict of interest.
